# Lifespan-regulating genes in *C. elegans*

**DOI:** 10.1038/npjamd.2016.10

**Published:** 2016-06-02

**Authors:** Masaharu Uno, Eisuke Nishida

**Affiliations:** 1 Department of Cell and Developmental Biology, Graduate School of Biostudies, Kyoto University, Kyoto, Japan

## Abstract

The molecular mechanisms underlying the aging process have garnered much attention in recent decades because aging is the most significant risk factor for many chronic diseases such as type 2 diabetes and cancer. Until recently, the aging process was not considered to be an actively regulated process; therefore, discovering that the insulin/insulin-like growth factor-1 signaling pathway is a lifespan-regulating genetic pathway in *Caenorhabditis elegans* was a major breakthrough that changed our understanding of the aging process. Currently, it is thought that animal lifespans are influenced by genetic and environmental factors. The genes involved in lifespan regulation are often associated with major signaling pathways that link the rate of aging to environmental factors. Although many of the major mechanisms governing the aging process have been identified from studies in short-lived model organisms such as yeasts, worms and flies, the same mechanisms are frequently observed in mammals, indicating that the genes and signaling pathways that regulate lifespan are highly conserved among different species. This review summarizes the lifespan-regulating genes, with a specific focus on studies in *C. elegans*.

## Introduction

Aging is an inevitable process in nearly all organisms, and the aging process was previously regarded as a passive entropic process of tissue deterioration caused by damage to macromolecules of the cell, such as genomic DNA, proteins and lipids. In the mid-nineteenth century, Darwin proposed that all wild species arise and develop through the natural selection of small inherited variations that increase the individual’s ability to survive and reproduce to ensure the welfare of the species. Because aging is a process that occurs after reproduction, biologists have presumed that the regulation of aging was not a critical factor for the evolution of life. However, hybrid mice generated from long- and short-lived animals inherit longevity,^1^ suggesting that lifespan is a genetically regulated trait. Since the isolation of the first long-lived mutant in *Caenorhabditis elegans* (*C. elegans*), a number of reports have described various genes and signaling pathways that regulate longevity in model organisms such as yeasts, worms, flies and mice.

Since Brenner first introduced the *C. elegans* model in the 1960s, this free-living soil nematode has been widely used as a model organism in many areas of research, including aging. *C. elegans* is a self-fertilizing hermaphrodite that lives for a few weeks when cultured at 20 °C, and it was first used as a model organism to study aging in the 1970s.^2–6^ In 1983, Klass reported a method for isolating longevity mutants in *C. elegans*, and it was used to isolate eight long-lived mutants for which an increased lifespan was most likely associated with reduced calorie intake.^7^ Johnson and his colleagues identified the long-lived mutant called *age-1*.^8,9^ Subsequent studies have led to the identification of the insulin/insulin-like growth factor-1 signaling (IIS) pathway as the first established lifespan-regulating signaling pathway.^10^ Since then, a number of genetic factors have been shown to have an important role in regulating the aging process, and these genetic factors may link environmental factors to the rate of aging. Here we summarize the biological factors associated with lifespan regulation, including signaling transduction pathways, epigenetic factors, sensory perceptions and other physiological processes.

## Lifespan-regulating genes

### Insulin/insulin-like growth factor-1 signaling

As previously mentioned, the first pathway implicated in the regulation of the aging process in animals was the IIS pathway.^10^ In *C. elegans*, mutations that decrease the activity of DAF-2, the *C. elegans* homolog of the insulin/insulin-like growth factor-1 receptor, more than double the lifespan of the animal,^11^ and mutations that affect the activity of the IIS downstream target AGE-1, the *C. elegans* homolog of phosphatidylinositol 3-kinase, are also associated with increased longevity.^8,9^ Longevity induced by a reduction in IIS signaling is entirely dependent on DAF-16, the *C. elegans* homolog of the forkhead box FoxO transcription factor.^11^ DAF-2, AGE-1 and DAF-16 constitute the three key components of the IIS pathway.^12–15^ Previous studies have demonstrated that modest inhibition of IIS promotes stress resistance and lifespan extensions in multiple species.^16–18^ Low IIS activity leads to the translocation of DAF-16 to the nucleus, where DAF-16 either activates or represses the genes involved in the cellular stress response (e.g., heat-shock proteins, superoxide dismutase and catalase), metabolism and autophagy. The combined effects of DAF-16-mediated transcriptional changes cause lifespan extension.^19–21^

Although DAF-16 has a pivotal role in regulating lifespan, several lines of evidence suggest that it does not necessarily act alone. First, the overexpression of DAF-16 in wild-type animals only slightly increases their lifespan, indicating that solely increasing levels of DAF-16 is not sufficient to significantly extend longevity.^22^ Second, the nuclear localization of DAF-16, which is necessary for its transcriptional activity, is not sufficient to extend lifespan.^23^ Third, although the canonical DAF-16-binding element^24^ is present in the 5-kb upstream region of 78% of *C. elegans* genes,^25^ only a small number of these genes are activated in young adult animals.^21^ Thus, there may be other factors that assist DAF-16 in activating particular genes in the appropriate context. Indeed, JNK-1 (the *C. elegans* homolog of c-Jun N-terminal kinase) and CST-1 (the *C. elegans* homolog of mammalian ste20-like kinase (MST)) have been shown to regulate DAF-16 activity via post-translational modification.^26,27^ Overexpression of JNK-1 or CST-1 promotes lifespan extension in a DAF-16-dependent manner,^26,27^ suggesting that JNK-1 and CST-1 stimulate DAF-16 activity. Lifespan extension induced by mutations in *daf-7* (a gene encoding a member of the *C. elegans* transforming growth factor-β family) is dependent on DAF-16, suggesting that the transforming growth factor-β pathway is an upstream regulator of IIS-mediated longevity.^28^ The ubiquitin proteasome system (UPS) also regulates DAF-16 activity. The loss of *rle-1*, a gene encoding an E3 ubiquitin ligase, increases lifespan,^29^ whereas the loss of *math-33*, a gene encoding deubiquitylase, suppresses the extended lifespan of *daf-2* mutants.^30^ There are several additional transcription factors that also function as cofactors of DAF-16. Similar to mutations in *daf-16*, mutations in *hsf-1* (a gene encoding the *C. elegans* homolog of heat-shock transcription factor), *skn-1* (a gene encoding the *C. elegans* homolog of nuclear respiration factor 2 (Nrf2)) or *pqm-1* (a gene encoding the C2H2-type zinc finger and leucine zipper-containing protein) suppress the lifespan extension phenotype of *daf-2* mutants.^31–33^ These findings suggest that HSF-1, SKN-1 and PQM-1 cooperate with DAF-16 to regulate the overlapping pro-longevity genes, although have the distinct target genes. SKN-1 has also been shown to be activated by MPK-1, the *C. elegans* homolog of ERK MAP kinase, and to regulate DAF-16 activity.^34^ It was recently reported that the transcription factor AP-1 collaborates with DAF-16 downstream of KGB-1 (one of the *C. elegans* JNK homologs) under fasting conditions.^35^ Although a number of additional genes that influence DAF-16 activity have been identified, the mechanisms by which these genes regulate the lifespan are not entirely understood.

*C. elegans* contains several tissue types, and the tissue-specific requirements of the IIS pathway with respect to longevity have been studied; however, a number of these studies have demonstrated conflicting results. Tissue-specific restoration experiments in *daf-2* and *age-1* mutants have revealed that the restoration of DAF-2 and AGE-1 expression in neurons, respectively, but not in the intestine is sufficient to decrease the lifespan of the long-lived *daf-2* and *age-1* mutants, respectively.^36^ However, a recent report demonstrated that AGE-1 expression in the intestine can decrease the long lifespan of the *age-1* mutant.^37^ Moreover, tissue-specific restoration experiments in a *daf-2;daf-16* mutant demonstrated that restoration of DAF-16 expression in the intestine extends the lifespan, whereas the restoration of DAF-16 expression in neurons exhibited only modest effects on lifespan.^38^ It has also been reported that the IIS pathway acts in both a cell-autonomous and non-autonomous manner,^36–40^ which might partly account for the complexity associated with lifespan regulation by the IIS pathway.

It has been reported that DAF-16 mediates the longevity effect associated with certain dietary restriction regimens. Although DAF-16 is dispensable for the longevity induced by chronic calorie restriction (e.g., *eat-2* mutant),^41^ it is required for the longevity induced by calorie restriction in middle-aged animals.^42^ DAF-16 is also required for intermittent fasting-induced longevity^43^ but not for continuous fasting-induced longevity.^44,45^ These results suggest that DAF-16 activation is dependent on a particular stimulus, as well as the timing or duration of the exposure to that stimulus.

### TOR signaling

TOR is a mechanistic target of rapamycin or mammalian target of rapamycin, and a serine/threonine kinase that regulates cell growth, proliferation, motility and survival, as well as protein synthesis, autophagy and transcription.^46^ TOR is activated under nutrient- and energy-sufficient conditions, which in turn stimulate growth and block salvage pathways, such as autophagy.^46^ Thus, a reduction in TOR activity is thought to mimic nutrient- and energy-deficient conditions. Consistent with this hypothesis, inhibiting TOR signaling increases the lifespan in *C. elegans* in a DAF-16-dependent manner.^47,48^ The effects on longevity induced by the inhibition of TOR signaling in *C. elegans* are mediated by the transcription factor PHA-4/FoxA.^49^ PHA-4 has been shown to regulate autophagy, a process that has a significant role in lifespan regulation.^50,51^ A combination of two lifespan-extending genetic manipulations, the inhibition of *daf-2* and inhibition of *rsks-1* (a *C. elegans* S6 kinase and a TOR target) additively extend the lifespan in *C. elegans.* ^52^ Thus, the IIS pathway and the TOR pathway act together to mediate the distinct manner in which lifespan is regulated in *C. elegans*.

It was recently reported that food restriction-induced TOR inhibition promotes longevity by inducing PHA-4.^43,53^ Interestingly, the TOR pathway promotes longevity by inhibiting the IIS pathway under fasting conditions.^43^ These observations suggest that the TOR pathway exerts both anti- and pro-longevity effects in a context-dependent manner.

### Sirtuin

Sirtuins are members of the nicotinamide adenine dinucleotide (NAD)-dependent protein deacetylase family of molecules, and directly linked to cellular nutrient signaling through NAD^+^.^54^ Sir2 is a positive regulator of lifespan in *Saccharomyces cerevisiae*,^55^ and in *C. elegans*, overexpression of SIR-2.1, the *C. elegans* homolog of Sir2, has been reported to extend lifespan^56^ in a DAF-16-dependent manner.^57^ SIR-2.1 has been reported to bind to DAF-16 in a 14-3-3-dependent manner in response to stress and promote DAF-16 activation.^57^ The pro-longevity activity of SIR-2.1 has been associated with a degree of controversy because a recent report demonstrated that the increased longevity, which was initially considered to result from *sir-2.1* overexpression, was caused by a background mutation in the transgenic animals used in these experiments.^58^ However, subsequent studies have demonstrated that the overexpression of *sir-2.1* in mutants outcrossed to a different genetic background significantly increased lifespan, albeit to a lesser extent than what was initially observed in the transgenic animals carrying the background mutation.^59–61^ Moreover, *sir-2.1* mutations suppress a variety of lifespan extension phenotypes, suggesting that sirtuins have a positive role in lifespan regulation.^60,62^

Studies have demonstrated that Sir2 can mediate mild dietary restriction-induced longevity^63^ but not severe dietary restriction-induced longevity^64^ in *S. cerevisiae*, although Tsuchiya *et al.* ^65^ reported that dietary restriction-induced longevity in *S. cerevisiae* is not dependent on sirtuins. In *C. elegans*, the increased longevity induced by SIR-2.1 overexpression and *eat-2*-induced longevity do not exhibit an additive effect,^66^ suggesting that SIR-2.1 has a role in dietary restriction-induced longevity similar to that of *eat-2*. However, *sir-2.1* has been shown to be dispensable for the increase in longevity induced by fasting and calorie restriction in middle-aged animals.^43–45,67^ Thus, sirtuins might exert pro-longevity effects under particular conditions of dietary restriction.

### AMP-activated protein kinase

AMP-activated protein kinase (AMPK) is a conserved cellular energy sensor that helps cells adapt to low-energy conditions.^68^ AMPK restores energy homeostasis by stimulating catabolic processes and blocking energy-consuming processes.^69^ The loss of *aak-2*, the gene encoding the *C. elegans* AMPK protein, decreases lifespan, and the increased expression of *aak-2* increases lifespan.^41^ Furthermore, overexpression of a constitutively active form of AMPK has been reported to increase lifespan to a greater extent than *aak-2* upregulation.^42,70^ Metformin is an indirect AMPK agonist that also extends lifespan in *C. elegans.* ^71^ AAK-2 is required for the longevity effects mediated by reduced IIS,^41^ and DAF-16 is required for the longevity effects mediated by activated AMPK, suggesting that DAF-16 and AMPK act together in a feedback loop. Indeed, AAK-2 activates DAF-16,^42^ and DAF-16 regulates the expression of *aakg-4*, a gene encoding a regulatory subunit of AMPK.^52^ Both AMPK and sirtuins are cellular energy sensors, as well as pro-longevity effectors, suggesting that these factors could interact with each other. Indeed, the increased longevity observed in animals overexpressing *sir-2.1* is dependent on *aak-2*,^72^ although these results were derived from experiments with transgenic worms harboring the confounding background mutation that was previously mentioned.^58^ CRTC (cAMP response element binding protein-regulating transcriptional coactivator) has been reported to mediate the lifespan extension induced by AMPK activation.^70^ Recently, neuronal AMPK activation was shown to regulate organismal lifespan via catecholamine signaling in *C. elegans.* ^73^

An AMPK–DAF-16 signaling pathway is activated by calorie restriction during middle age and mediates lifespan extension.^42^ However, this pathway is dispensable to lifespan extensions induced by chronic calorie restriction, such as that observed in the *eat-2* mutant.^47^ Moreover, the transcription factors that are required for lifespan extension induced by chronic calorie restriction (e.g., PHA-4 and SKN-1) are dispensable for longevity induced by calorie restriction in middle-aged animals.^67^ Thus, the timing and quantity of food might be important factors in specifying which signaling pathway is activated.

### Mitochondria

Mitochondria are essential cell organelles that provide functions that are central to cellular metabolism and apoptosis. Mitochondria are also a major source of reactive oxidative species (ROS). ROS are responsible for damage to macromolecules such as DNA, protein and lipids, and they also promote the deterioration of cells, tissues and, ultimately, the entire organism. Mitochondrial ROS are thought to be the primary cause of aging, and this hypothesis is referred to as the mitochondrial free radical theory of aging. Indeed, non-biased large-scale RNAi screens for genes associated with lifespan extension have revealed that most of the genes that promote lifespan extension are involved in metabolic pathways and components of the mitochondrial electron transport chain.^74–77^ These comprehensive analyses have also demonstrated that mitochondria have an important role in aging. However, the SOD-2 (*C. elegans* mitochondrial superoxide dismutase) loss-of-function mutation, which is predicted to increase oxidative damage, extends lifespan.^78^ The same research group demonstrated that treatment with the pro-oxidant paraquat also extended the lifespan of *C. elegans,* ^79^ suggesting that mild mitochondrial stress renders organisms less susceptible to subsequent perturbations. Furthermore, inhibiting respiration can extend lifespan by inducing a moderate increase in ROS, and this effect is mediated by HIF-1, AAK-2,^80^ CHE-23,^81^ CEP-1^82^ and SKN-1.^83^ Moreover, the intrinsic apoptosis axis has also been shown to mediate longevity.^84^

Because mitochondria have a central role in cellular energy production, it is plausible that mitochondria also have an important role in regulating lifespan in response to dietary restrictions. Indeed, lifespan extension by glucose restriction is thought to be mediated by mitohormesis (the specific type of hormesis that is caused by increased formation of ROS within the mitochondria) in *C. elegans.* ^85^ Restricting glucose promotes the formation of ROS, which in turn may elicit the mitohormesis response.^85^ SKN-1 and AAK-2 mediate both calorie restriction-induced longevity^42,86^ and mitohormesis-mediated longevity,^80,83^ suggesting that SKN-1 and AAK-2 might function as a link between dietary restriction and mitochondria function.

### Epigenetic mechanisms

Epigenetic mechanisms, such as DNA methylation, histone modification and noncoding RNAs, regulate the interpretation of genetic information and are linked to numerous biological processes. In humans, epigenetic changes correlate with older age in normal individuals and are a hallmark of patients with progeria syndrome; however, the significance of epigenetics in regulating the rate of aging is not well understood.

#### Histone-modifying enzymes

It has been reported that histone-modifying enzymes also have a role in lifespan regulation. A genome-wide RNAi screen identified two SET domain proteins (SET-9 and SET-15) that accelerate aging.^76^ Greer *et al.* ^87^ demonstrated that inhibition of the histone H3K4 methylation complex (composed of ASH-2, WDR-5 and SET-2) and overexpression of RBR-2, the enzyme that mediates H3K4 demethylation, resulted in lifespan extension. The lifespan extension associated with H3K4-modifying enzymes is dependent on the production of mature eggs.^87^ Surprisingly, although these genetic manipulations were present only in the parent animals, up to four generations of descendants presented extended lifespans.^88^ However, the molecular mechanisms for the transgenerational inheritance of longevity remain unknown. In addition, the inhibition of H3K27 demethylase UTX-1 has also been shown to extend lifespan.^89,90^

#### MicroRNA

Noncoding RNAs also have an important role in lifespan regulation. MicroRNA (miRNA) is a class of noncoding RNA molecules that regulate the expression of target mRNAs in a sequence-dependent manner. The miRNA *lin-4* was the first discovered miRNA, and it regulates the lifespan of *C. elegans.* ^91^ Lifespan regulation associated with *lin-4* and its target LIN-14 is mediated by the IIS pathway.^91^ Following the identification of *lin-4*, a number of additional miRNAs have been implicated in lifespan regulation.^92^ Recently, a long noncoding RNA named *tts-1* was shown to regulate lifespan by modulating ribosomal activity.^93^

Although dietary restrictions and epigenetic mechanisms are both known to be involved in lifespan regulation, the potential links between epigenetics and dietary restriction-induced longevity remain largely unknown. *miR-228* and *miR-71* were recently reported to be necessary for calorie restriction-induced longevity.^94^ These miRNAs regulate lifespan by interacting with the dietary restriction responsive transcription factors PHA-4/FoxA and SKN-1/Nrf2.^94^ Pandit *et al.* ^95^ demonstrated that PHA-4 regulates the expression of miRNAs in calorie-restricted animals. These studies suggest that miRNAs are involved in at least some of the mechanisms that mediate dietary restriction-induced longevity.

### Proteostasis

Protein homeostasis, or proteostasis, is essential to life. The loss of proteostasis is often involved in protein aggregation, a cellular process that is associated with many age-related disorders and often observed in aged organisms.^96^ The loss of proteostasis results in the deterioration in cellular function; therefore, it may be one of the main causes of organismal aging. Proteostasis is maintained by a complex interplay among protein synthesis, degradation and quality control. Polysome profiling analyses have revealed that the rate of protein synthesis in *C. elegans* is markedly decreased in 10-day-old animals compared with 4-day-old animals.^97^ These findings suggest that the loss of proteostasis during old age does not necessarily result from excess protein synthesis. Paradoxically, the *C. elegans* lifespan can be extended by decreasing protein synthesis. The long-lived *daf-2* mutant exhibits a reduced rate of protein translation.^93^ The inhibition of eukaryotic initiation factors, ribosomal proteins and TOR signaling molecules, which is responsible for decreased translation in these animals, has been reported to account for the increased lifespan.^98–100^ The protein degradation pathway is also used as a method for reshaping proteome function. The UPS and autophagy are major protein degradation mechanisms. One study used *in vivo* imaging techniques (chimeric green fluorescent protein fused to a non-cleavable ubiquitin moiety) to demonstrate that UPS activity declines soon after animals complete development.^101^ Moreover, an increase rather than a decrease in UPS activity is associated with lifespan extension.^29,30,35,102^ These results suggest that decreased UPS activity contributes to the collapse of proteostasis in old age. Autophagy is another cellular process that has an important role in lifespan regulation. The protein quality control system, which consists of the heat-shock response, the unfolded protein response (UPR) of the endoplasmic reticulum (ER) (UPR^ER^) and the mitochondrial UPR (UPR^mt^), is also important for proteostasis. The various genes involved in the protein quality control system are regulated by designated transcription factors (HSF-1 for heat-shock response, XBP-1 and ATF6 for UPR^ER^, and ATFS-1 for UPR^mt^).^96^ Animals utilize these protein quality control pathways to manage a wide range of acute and chronic stress conditions during the aging process.^96^ The activation of heat-shock response or UPR^ER^ by overexpression of *hsf-1* or *xbp-1*, respectively, extends lifespan,^101,103^ suggesting that heat-shock response and UPR^ER^ also have a role in lifespan regulation. Although the longevity induced by the inhibition of respiration requires UBL-5, a coactivator of UPR^mt^,^104^ the activation of UPR^mt^ does not always extend lifespan.^105^ Thus, although the link between longevity and UPR^mt^ is plausible, it is still not clear whether the activation of UPR^mt^ alone is sufficient for lifespan extension.

Autophagy is an evolutionarily conserved intracellular degradation system that delivers cytoplasmic components to the lysosome.^106^ The autophagy system has been shown to be involved in several longevity pathways. The first study to evaluate the role of autophagy in lifespan regulation in *C. elegans* revealed that RNAi targeting *bec-1*, a gene encoding the *C. elegans* ortholog of yeast ATG6, significantly suppresses the long lifespan of *daf-2* mutants.^20^ Subsequent studies have demonstrated that autophagy genes are also required for the longevity induced by reduced IIS and indicated that autophagy is induced in *daf-2* Dauer larvae and adults.^20,50,107,108^ However, one study reported that RNAi targeting autophagy genes can extend lifespan under certain conditions.^109^ In addition, autophagy can regulate calorie restriction-induced lifespan extensions. Accordingly, RNAi targeting autophagy genes abolishes the lifespan extension induced by the *eat-2* mutation or TOR signaling inhibition.^50,108^ Moreover, autophagy activity is enhanced in both TOR RNAi-treated animals and animals subjected to dietary restriction in a PHA-4-dependent manner.^50^ Autophagy also facilitates lipid storage in the intestine.^110^ Thus, autophagy might provide energy to the animal by stimulating lipogenesis in response to dietary restrictions. Consistent with this hypothesis, fasting induces both lipase genes and autophagy genes via activation of the HLH-30 transcription factor.^110^ Inhibition of mitochondrial respiration extends lifespan in wild-type animals but not in autophagy mutants,^108^ suggesting that autophagy is also required for the longevity induced by the inhibition of mitochondrial respiration. A recent report demonstrated that mitophagy, a selective type of autophagy targeting mitochondria for degradation, has an important role in maintaining mitochondrial homeostasis during aging.^111^ Together, these observations indicate that multiple lifespan-extending pathways require autophagy.

### Hypoxia inducible factor-1

Hypoxia inducible factor 1 (HIF-1) is considered to be the master transcriptional regulator mediating the cellular response to hypoxia.^112^ HIF-1 is degraded by the E3 ubiquitin ligase von Hippel Lindau under normal oxygen conditions, and HIF-1 is stabilized under low-oxygen conditions.^112^ Stabilization of HIF-1 by the downregulation of von Hippel Lindau-1 or overexpression of HIF-1 significantly increases lifespan.^113,114^ Consistent with these observations, hypoxia induces lifespan extension in a HIF-1-dependent manner.^115^ HIF-1 also mediates the lifespan extension induced by the increase in ROS following paraquat treatment or respiration inhibition.^80,116^ Neuronal HIF-1 was recently demonstrated to mediate lifespan extensions by regulating the expression of intestinal flavin-containing monooxygenase (*fmo-2*) via serotonin secretion.^117^

## Lifespan regulation by the interplay of different tissues

### Gonads

**Figure 1:**
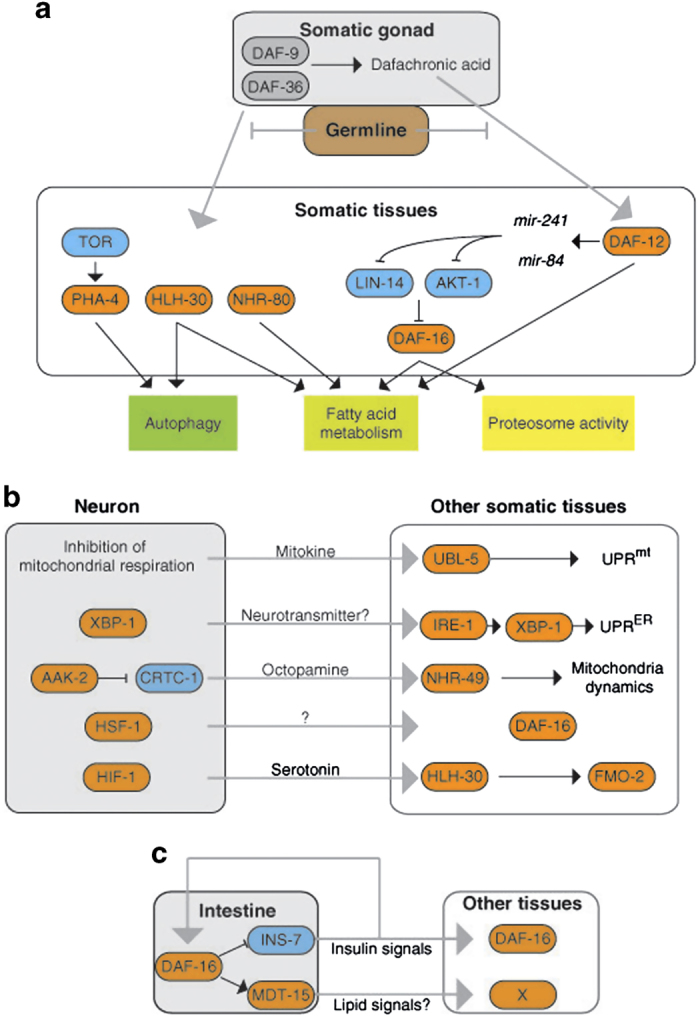
Tissue interplay that regulates lifespan. (**a**) Genes that mediate germline elimination-induced lifespans in *C. elegans*. Germline elimination enhances the steroid signal dafachronic acid, which is produced in somatic gonads. DAF-12 regulates the expression of *mir-84* and *mir-241*, and increased *mir-84* and *mir-241* expression results in the downregulation of two DAF-16 inhibitors (AKT-1 and LIN-14) and promotes DAF-16 nuclear localization and activation. Germline elimination also activates the transcription factors PHA-4, HLH-30 and NHR-80. The activation of these transcription factors leads to changes in fatty acid metabolism, as well as enhanced autophagy and proteasome activity. (**b**) The genetic manipulations in neuronal cells are sufficient for increasing organismal lifespan. The RNAi of *cco-1* or overexpression of *xbp-1*, *aak-2*, *hsf-1* or *hif-1* extends lifespans by regulating other tissues. (**c**) Intestinal DAF-16 regulates both DAF-16 and a non-DAF-16 transcription factor (referred to as X here) in other tissues via INS-7- and MDT-15-dependent lipid signals, respectively.

The gonad consists of the germline and somatic gonad, and it has an important role in regulating lifespan in *C. elegans.* ^118^ The removal of germline cells by laser microsurgery or genetic manipulation (e.g., *glp-1* mutants) increases lifespan, whereas the removal of the gonad (both the germline and somatic gonad) does not.^119,120^ Because the ablation of both the germline and somatic gonad results in sterility, the longevity induced by germline elimination is not only solely related to a resource trade-off but also to endocrine-mediated lifespan regulation (Figure 1a). In fact, certain endocrine signaling pathways have been shown to regulate longevity in animals without germlines^118^ (Figure 1a).

The nuclear hormone receptor DAF-12, which responds to the dafachronic acid (DA) ligand, is a key regulator of germline elimination-induced longevity.^119^ DAF-9 and DAF-36 are also components of the steroid hormone-signaling pathway that contributes to gonad-associated longevity.^121–123^ DA supplementation restores the longevity triggered by ablation of both germline and somatic gonads in a DAF-12-dependent manner, suggesting that the somatic gonad is involved in DA production.^124^ However, the location of DA production is not important because the overexpression of DAF-9 in other tissues is sufficient to restore the longevity of animals lacking gonads.^124^ The tissues in which DAF-12 functions to increase lifespan have yet to be determined. DAF-16 is an effector of the IIS pathway and a critical regulator of germline elimination-induced longevity.^119^ The DA–DAF-12 axis regulates DAF-16 nuclear localization and activity.^123,125^ DAF-12 regulates the expression of *mir-84* and *mir-241*, and the increased expression of these two miRNAs leads to the downregulation of two inhibitors of DAF-16 (AKT-1 and LIN-14), thereby promoting DAF-16 nuclear localization and activation.^126^ DAF-12 also regulates the expression of the fatty acid reductase *fard-1* and the lipase *lips-17.* ^127^ DAF-16 regulates the expression of the lipase *lipl-4* ^128^ and *rnp-*6, a subunit of the 19S proteasome.^129^ The upregulation of these genes contributes to germline elimination-induced longevity.

Germline elimination-induced longevity requires other transcription factors in addition to DAF-12 and DAF-16, including NHR-80 and PHA-4.^51,130^ NHR-80 links FAT-6 (a gene encoding an acyl-CoA desaturase)-induced fatty acid desaturation to longevity in germline-less animals in a DAF-16-independent manner.^130^ TOR expression is reduced in *glp-1* mutants and results in enhanced autophagy in the intestine via the activity of PHA-4.^51^ It should be noted that germline elimination regulates DAF-16 in a manner distinct from that of the IIS pathway. Although reduced IIS induces DAF-16 nuclear translocation in all tissues,^22^ germline elimination induces DAF-16 nuclear translocation only in the intestine of young adult animals.^125^ In summary, germline elimination extends lifespan by regulating several signaling pathways in somatic tissues, such as the intestine, via endocrine pathways.

### Somatic tissues

Animals sense and process environmental signals to help them prepare for environmental changes. Lifespan regulation by sensory perception was initially identified in *C. elegans* in studies using mutants defective in sensory perception,^131^ and the longevity of these mutants was largely but not entirely dependent on DAF-16.^131^ Gustatory, olfactory and thermosensory neurons were all subsequently shown to influence lifespan.^132,133^ Together, these studies suggest that neurons that sense environmental cues influence lifespan. Thus, certain sensory neurons shorten lifespan and others extend lifespan in an environmental context-dependent manner.^133–137^ Food availability is one of the most important environmental factors that influence lifespan. Indeed, certain mutants with defective sensory neurons exhibit a lifespan phenotype only when they eat certain bacteria as food.^134^ Interestingly, diffusible bacterial products suppress lifespan extension by promoting fasting,^138^ suggesting that not only food consumption but also food sensing is important for lifespan regulation. In addition, neuronal SKN-1 has been shown to mediate dietary restriction-induced longevity.^86^ Recent studies have demonstrated that genetic manipulations in neuronal cells only are sufficient to increase organismal lifespan (Figure 1b). The inhibition of respiration by neuron-specific *cco-1* RNAi is sufficient to increase lifespan, and it induces the intestinal UTR^mt^ response,^104^ suggesting that a systemic factor secreted from neurons might regulate global cellular responses. The overexpression of active AAK-2 or stable HIF-1 in neurons extends lifespan via the biogenic monoamine neurotransmitter serotonin or octopamine, respectively.^73,117^ The overexpression of a spliced form of XBP-1 in neurons extends lifespan in a neurosecretion-dependent manner,^101^ and the overexpression of HSF-1 increases thermotolerance and lifespan.^103^ The thermosensory circuit is required for the increase in thermotolerance induced by HSF-1 overexpression but not for the lifespan extension effect.^103^ However, intestinal DAF-16 is required for the lifespan extension induced by HSF-1 overexpression but not for the increase in thermotolerance.^103^ These observations suggest that neurons might be a key source of extracellular signals that regulate organismal lifespan.

The intestine has an important role in regulating organismal lifespan. There are several lines of evidence suggesting that the intestine receives signals from other cells that modulate organismal lifespan. First, intestinal restoration of DAF-16 significantly increases the lifespan of *daf-2;daf-16* mutants.^38^ Second, intestinal DAF-16 is required for the lifespan extension induced by neuronal HSF-1 overexpression.^103^ Third, an increase in intestinal *fmo-2* expression is required for neuronal HIF-1 activation.^117^ Fourth, intestinal UPR^mt^ or UPR^ER^ is induced in response to neuronal *cco-1* knockdown or the overexpression of spliced *xbp-1*, respectively.^101,103^ These observations suggest that the intestine is a key target organ responsible for regulating lifespan at the organismal level. Intestinal DAF-16 regulates both DAF-16 and a non-DAF-16 transcription factor in other tissues via INS-7-^139^ and MDT-15-dependent lipid signals,^40^ respectively, suggesting that the intestine can act as a sender tissue as well as a receiver tissue (Figure 1c).

### Conclusions and future challenges

Although genetic factors have important roles in the regulation of organismal lifespans, environmental factors are also important in lifespan regulation. Following the groundbreaking studies that first identified lifespan-regulating genes in the 1980s and 1990s, a large number of genes have been shown to regulate organismal lifespan. Thus, one of the next major challenges is to determine how these genes link environmental factors to lifespan regulation. As previously mentioned, the relationship between genes and diet is the most important lifespan-regulating environmental cue, and it has been examined in many organisms. Environmental temperature and oxidative status are additional environmental cues that influence lifespan,^4,79^ and studies examining the relationship between genes and these specific environmental cues have recently been initiated.

One of the goals of aging research is to not only extend lifespan but also extend healthspan. Worms display certain age-associated characteristics that resemble those observed in humans;^140^ therefore, worms also serve as a useful model for healthspan studies. Healthspan studies in worms include those that have examined mobility declines^141^ and pharyngeal pumping,^142^ fluorescent compound dynamics (including lipofuscin and advanced glycosylation end products),^143,144^ and neuromuscular changes.^141,145,146^ In addition, the identification of small molecules that potentially slow down aging and extend lifespan in multiple species is another major challenge and goal associated with aging research. *C. elegans* has multiple advantages that make it a useful model for identifying and evaluating chemical compounds that extend lifespan.^147^

Aging induces declines in the integrity and function of tissues throughout the organismal body. If aging affects each tissue independently, the mechanism underlying organismal aging could be elucidated by dissecting the aging process associated with each cell type. If certain tissues have a role in coordinating the aging process among different tissues, researchers might focus on the systemic factors used by these tissues to communicate with other tissues. There are several lines of evidence for the presence of an aging control center. Thus, the location and mechanism associated with the regulation of organismal aging by lifespan-regulating genes are additional issues that merit further investigation. These studies will facilitate the development of therapeutic strategies that target the aging control center to help promote health spans in humans.
